# Targeting CD177: A Novel Therapeutic Strategy for NLRP3-Associated Autoinflammatory Diseases

**DOI:** 10.3390/ijms27062841

**Published:** 2026-03-20

**Authors:** Yinghua Zhu, Fangfang Zhang, Siping Li, Zhihua Tian, Zaixue Jiang, Fen Lv, Xiaomei Zeng, Zhongjun Zhou, Baimao Zhong, Qi Peng, Xiaomei Lu

**Affiliations:** 1 Department of Genetic Medicine, Dongguan Children’s Hospital Affiliated to Guangdong Medical University, Dongguan 523325, China; 2 Key Laboratory for Children’s Genetics and Infectious Diseases of Dongguan, Dongguan 523323, China; 3 Department of Medical and Molecular Genetics, Dongguan Institute of Pediatrics, Dongguan 523325, China; 4 School of Biomedical Sciences, LKS Faculty of Medicine, The University of Hong Kong, Hong Kong SAR, China

**Keywords:** autoinflammatory diseases, CD177, neutrophil, siRNA therapy, IL-1β refractoriness

## Abstract

NLRP3-associated autoinflammatory diseases (NLRP3-AIDs) are rare autoinflammatory disorders caused by uncontrolled inflammasome activation. While IL-1β blockade is first-line therapy, many patients respond inadequately, highlighting a need for alternative strategies. Transcriptomic analysis was performed on immune cells from a patient with an NLRP3 L573W mutation. Functional validation of CD177 as a downstream effector of NLRP3 activation was conducted. A novel NLRP3 L573W knock-in mouse model was established. Correlation between CD177 expression, disease severity, neutrophilia, and tissue damage was assessed. Therapeutic efficacy of siRNA-mediated CD177 silencing was evaluated and compared with IL-1β blockade. CD177, a neutrophil-specific protein, was significantly upregulated in NLRP3-mutant cells and confirmed as a direct downstream effector of NLRP3 activation. The NLRP3 L573W knock-in mouse recapitulated human disease heterogeneity, from mild self-limited inflammation to severe multi-organ pathology. CD177 expression correlated with disease severity, neutrophilia, and tissue damage. siRNA-mediated CD177 silencing attenuated systemic inflammation, reduced neutrophil infiltration and cytokine levels (IL-1β, IL-6, TNFα), and ameliorated multi-organ damage, with effects comparable to or exceeding those of IL-1β blockade. CD177 is a non-canonical amplifier of NLRP3-driven inflammation. Targeting CD177 represents a superior therapeutic strategy for NLRP3-AIDs, including IL-1β-refractory cases.

## 1. Introduction

*NLRP3*-associated autoinflammatory diseases (NLRP3-AIDs) are a group of rare, inherited disorders characterized by uncontrolled systemic inflammation due to gain-of-function mutations in the *NLRP3* gene [1,2,3]. These mutations lead to the constitutive activation of the *NLRP3* inflammasome, resulting in the caspase-1-dependent overproduction of interleukin-1β (IL-1β) and other pro-inflammatory cytokines [4,5]. The clinical spectrum of *NLRP3*-AIDs includes conditions such as familial cold autoinflammatory syndrome (FCAS), Muckle–Wells syndrome (MWS), and chronic infantile neurological cutaneous articular syndrome (CINCA/NOMID), manifesting with fever, urticarial rash, arthralgia, and in severe cases, progressive sensorineural hearing loss and central nervous system involvement [6,7].

Targeting the IL-1 pathway has revolutionized the management of *NLRP3*-AIDs, with IL-1β antagonists such as canakinumab serving as the first-line therapy [8,9,10,11]. However, a significant clinical challenge remains: approximately 20% of patients exhibit a suboptimal or poor response to IL-1 blockade [12]. This treatment failure may stem from the complex inflammatory network in which other cytokines (e.g., IL-6, TNFα) and immune cells contribute to disease pathogenesis independently of IL-1β [13]. Additionally, some patients may develop a reduced response to IL-1 inhibitors over time, indicating potential drug tolerance or secondary resistance [14,15]. These limitations underscore the critical need to identify novel molecular drivers downstream of *NLRP3* activation that could serve as alternative therapeutic targets for non-responders.

Neutrophils are pivotal effector cells in *NLRP3*-AIDs, dominating the inflammatory infiltrate and amplifying tissue damage [16,17]. Yet, the specific mechanisms by which hyperactive *NLRP3* signaling dysregulates neutrophil function beyond IL-1β release remain poorly defined. Identifying neutrophil-specific factors that are directly upregulated by *NLRP3* mutations could provide crucial insights into disease pathogenesis and reveal new therapeutic opportunities.

CD177 is a glycosylphosphatidylinositol (GPI)-anchored glycoprotein exclusively expressed on the surface of neutrophils and a subset of monocytes [18,19]. It plays roles in neutrophil transendothelial migration, respiratory burst, and modulation of inflammatory responses, although its precise functions are still being elucidated [19].

Neutrophils, the most abundant type of white blood cells, are produced in the bone marrow and are among the first responders to sites of infection or inflammation [20]. Their primary functions include phagocytosis, degranulation, and the formation of neutrophil extracellular traps (NETs) to eliminate pathogens [21]. However, their excessive or dysregulated activation can also cause significant host tissue damage, a hallmark of many inflammatory diseases including NLRP3-AIDs.

In our previous work, our research team identified a pediatric patient harboring a heterozygous NLRP3 mutation (c.1718T > G, p.L573W) [22]. In the present study, transcriptomic profiling of peripheral blood cells from this patient revealed a marked upregulation of CD177, a neutrophil-specific surface protein, which emerged as one of the most significantly upregulated genes. While this finding was based on a single patient, it provided a crucial starting point for our mechanistic investigation. We hypothesized that CD177 acts as a key downstream effector of *NLRP3* mutations, driving inflammatory pathology. Using a novel murine model of the *NLRP3* L573W mutation that recapitulates the phenotypic heterogeneity of the human disease, we validated the correlation between CD177 expression and disease severity. Furthermore, we demonstrated that targeting CD177 with siRNA not only ameliorates systemic inflammation and multi-organ damage but also exerts effects comparable to IL-1β blockade in our preclinical model. Our work establishes CD177 as a novel diagnostic biomarker and a promising therapeutic target for NLRP3-AIDs, particularly for patients refractory to current IL-1-targeted therapies.

## 2. Results

### 2.1. CD177 Is Significantly Upregulated in Patients with NLRP3-AID and Is a Specific Downstream Effector of NLRP3 Activation

**Figure 1:**
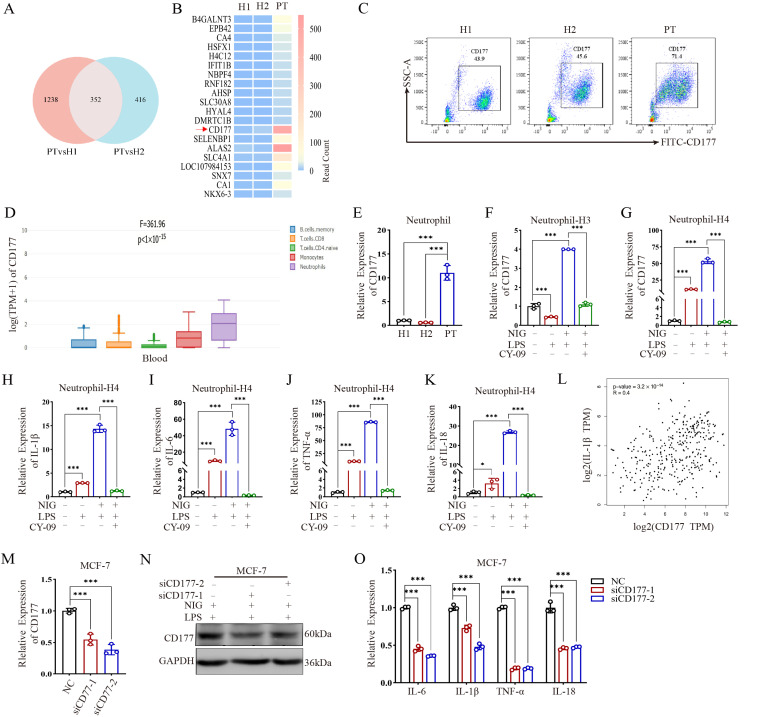
CD177 is elevated in patients with NLRP3-AID. (**A**) Venn diagram shows the results of the transcriptome sequencing analysis. (**B**) Heatmap shows differentially expressed genes. (**C**) Flow cytometry detects the expression of CD177 in peripheral blood of NLRP3 mutant patient (PT) and healthy controls (H). (**D**) GEPIA analysis of the expression of CD177 in different immune cells in peripheral blood. (**E**) qPCR assay detects CD177 expression in peripheral blood neutrophils. (**F**,**G**) qPCR assay detects the expression of CD177 in neutrophils from two healthy controls (H3 and H4) treated with NLRP3 agonist (Nigericin) or NLRP3 inhibitor (CY-09). (**H**–**K**) qPCR assay detects the expression of IL-1β, IL-6, TNFα and IL-18 in neutrophils from HCs treated with Nigericin or CY-09. (**L**) GEPIA analysis of the expression correlation between CD177 and IL-1β. (**M**) qPCR assay examines the expression of CD177 in MCF-7 cells transfected with CD177 siRNA or negative control siRNA. (**N**) Western blot analysis of CD177 protein levels in MCF-7 cells transfected with CD177 siRNA or negative control siRNA. (**O**) The mRNA levels of IL-1β, IL-6, TNFα and IL-18 in MCF-7 cells transfected with CD177 siRNA or negative control siRNA. qPCR Data are shown as mean ± s.d. Student’s *t*-test was used. Statistical significance was determined as * *p* < 0.05; *** *p* < 0.001.

To investigate the molecular mechanism underlying *NLRP3* L573W-induced autoinflammation, we performed transcriptomic sequencing on peripheral blood immune cells from a patient and matched healthy controls (HCs). This analysis revealed 352 significantly upregulated genes. Among these, CD177 emerged as one of the most markedly elevated transcripts (Figure 1A,B). Transcriptomic analysis also identified several downregulated genes (e.g., B4GALNT3, EPB42, CA1), but we focused on CD177 due to its known neutrophil-specific expression and potential role in inflammation, making it a compelling candidate for further investigation in the context of NLRP3-AID. Flow cytometry analysis confirmed a substantial increase in CD177 protein expression in the patient’s peripheral blood cells compared to HCs (Figure 1C). Interrogation of the GEPIA database indicated that CD177 is predominantly expressed in myeloid lineages, particularly neutrophils (Figure 1D), a finding corroborated by qPCR showing elevated CD177 mRNA in patient-derived neutrophils (Figure 1E). In Figure 1E, H1 and H2 refer to Healthy Control 1 and Healthy Control 2, respectively.

To establish a direct causal link between NLRP3 signaling and CD177 expression, we treated Lipopolysaccharide (LPS) and neutrophils from healthy donors with the NLRP3 agonist nigericin or the inhibitor CY-09. LPS and Nigericin stimulation significantly promoted CD177 expression, an effect that was potently reversed by CY-09 pre-treatment (Figure 1F,G), indicating that CD177 upregulation is a specific consequence of NLRP3 pathway activation. The difference in fold-change magnitude between Figure 1F,G is likely due to interindividual variability among healthy donors (HC3 vs. HC4) and differences in baseline CD177 expression. As expected, NLRP3 activation also drove the expression of canonical inflammatory cytokines (IL1B, IL6, TNF, IL18), which were suppressed by inhibition (Figure 1H–K).

Public database analysis revealed a moderate positive correlation between CD177 and IL1B expression (Figure 1L). To functionally test if CD177 can regulate cytokine production, we employed an in vitro MCF-7 cell model. Although MCF-7 cells do not endogenously express high levels of CD177, they provide a tractable system to assess the specific impact of CD177 modulation without the confounding background of potent innate immune pathways present in primary neutrophils. siRNA-mediated knockdown of CD177 in these cells successfully reduced its mRNA and protein levels (Figure 1M,N) and, importantly, significantly downregulated the expression of IL1B, IL6, TNF, and IL18 (Figure 1O). These data posit CD177 not only as a diagnostic biomarker for *NLRP3*-AID but also as a potential key driver of the associated inflammation.

### 2.2. A Novel NLRP3 L573W Mutant Mouse Model Recapitulates the Phenotypic Heterogeneity of Human Disease

**Figure 2:**
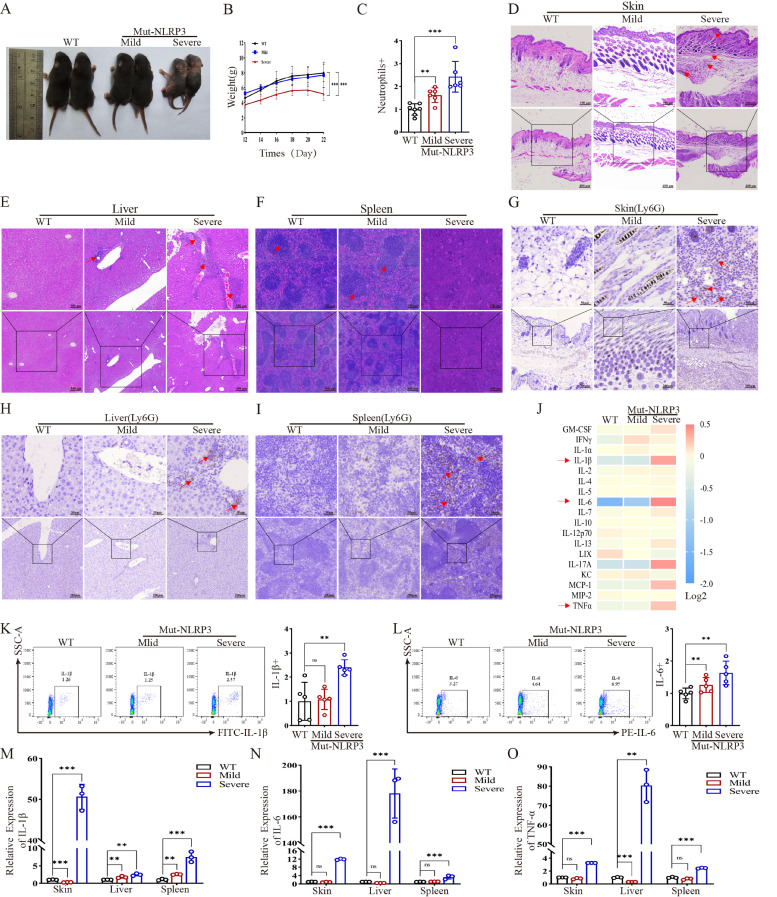
NLRP3 mutant mice exhibit phenotypic heterogeneity despite identical genotypes. (**A**) Images of wild-type (*Nlrp3*^+/+^) mice and NLRP3 mutant (*Nlrp3*^L573W/+^) mice with different inflammatory states. Notably, both mild and severe phenotypic groups are confirmed to be heterozygous for the p.L573W mutation. (**B**) Body weight of wild-type mice, NLRP3 mutant (Nlrp3 L573W/+) mice with different inflammatory states (weight measured starting from day 12, every other day). (**C**) Flow cytometry analysis of neutrophils in mouse peripheral blood (*n* = 6 per group). (**D**–**F**) Histopathological detection of mouse tissues (skin, liver, spleen) showing varying degrees of inflammatory infiltration, Scale bars: 400 μm, 100 μm. (**G**–**I**) Immunohistochemical detection of mouse tissues (skin, liver, spleen) showing differences in neutrophil marker Ly6G expression, Scale bars: 200 μm, 50 μm. (**J**) Cytokine microarray in serum of wild-type mice and NLRP3 mutant mice with different inflammatory states. (**K**,**L**) Flow cytometry detection of IL-1β and IL-6 protein expression in mouse peripheral blood cells (*n* = 6 per group). (**M**–**O**) qPCR detection of IL-1β, IL-6, and TNFα mRNA expression levels in mouse skin, liver, and spleen tissues (*n* = 3). qPCR Data are shown as mean ± s.d. Student’s *t*-test was used. Statistical significance was determined as ** *p* < 0.01; *** *p* < 0.001; ns, not significant. CD177 expression is elevated in *NLRP3* mutant mice and correlates with disease severity.

To functionally validate the role of CD177 in vivo, we generated a mouse model harboring the heterozygous *NLRP3* L573W mutation. Mirroring the clinical heterogeneity in patients, the mutant mice exhibited two distinct phenotypes: a mild inflammatory group that showed transient skin inflammation and spontaneous recovery, and a severe inflammatory group characterized by stunted growth, persistent severe dermatitis, and multi-organ involvement (Figure 2A,B). The basis for the mild versus severe phenotypes is currently unknown and may involve stochastic or environmental factors; no consistent correlation with sex or exact age at analysis was observed. Hematological analysis revealed significant neutrophilia in the peripheral blood of all mutant mice, with counts significantly higher in severe mice compared to both mild mutants and wild-type (WT) controls (Figure 2C). Histopathological examination of severe mice showed pronounced skin hyperplasia, dermal inflammatory infiltrates, hepatic vacuolar degeneration with portal inflammation, and disrupted splenic architecture with expansion of the red pulp (Figure 2D–F). Immunohistochemistry confirmed extensive infiltration of Ly6G+ neutrophils in these organs (Figure 2G–I).

Consistent with the histopathological severity, cytokine profiling of serum revealed a robust increase in IL-6, IL-1β, MCP-1, and TNFα in severe mice, with IL-6 being the most elevated. Mild mice showed only a modest increase in IL-6 and IFNγ (Figure 2J). Flow cytometry and qPCR analyses validated the marked elevation of IL-1β and IL-6 in the blood and tissues (skin, liver, spleen) of severe mice, but not in mild mice (Figure 2K–O). This model effectively recapitulates the spectrum of human *NLRP3*-AID, providing a robust platform for pathogenetic and therapeutic investigation.

**Figure 3:**
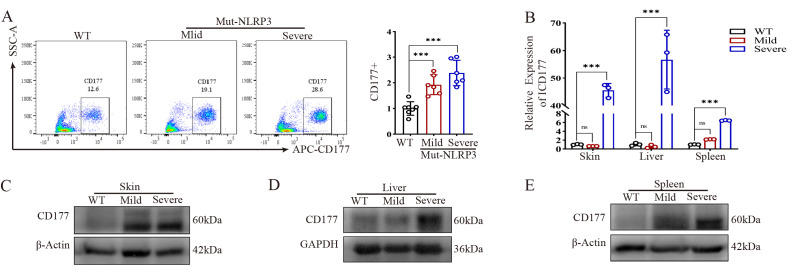
CD177 expression is upregulated in NLRP3 mutant mice. (**A**) Flow cytometry detection of CD177 protein expression in mouse peripheral blood (*n* = 6). (**B**) qPCR detection of CD177 mRNA expression levels in mouse skin, liver, and spleen tissues (*n* = 3). (**C**–**E**) Western blot analysis of CD177 protein expression levels in mouse skin, liver, and spleen tissues. qPCR Data are shown as mean ± s.d. Student’s *t*-test was used. Statistical significance was determined as *** *p* < 0.001; ns, not significant.

We next assessed whether CD177 expression was also dysregulated in our murine model. Flow cytometry analysis confirmed that CD177 protein was significantly elevated in the peripheral blood of severe mutant mice, with a more modest increase in mild mutants compared to WT mice (Figure 3A). Figure 3A shows CD177 expression specifically in neutrophils (Ly6G+ cells) from mouse peripheral blood. Furthermore, Cd177 mRNA and protein levels were significantly higher in the skin, liver, and spleen of severe mice compared to both WT and mild mice (Figure 3B–E). This strong correlation between CD177 expression levels and inflammatory severity suggests its involvement in mediating the pathological process.

### 2.3. IL-6 Neutralization Shows Limited Efficacy in Alleviating Systemic Inflammation in Severe NLRP3 Mutant Mice

**Figure 4:**
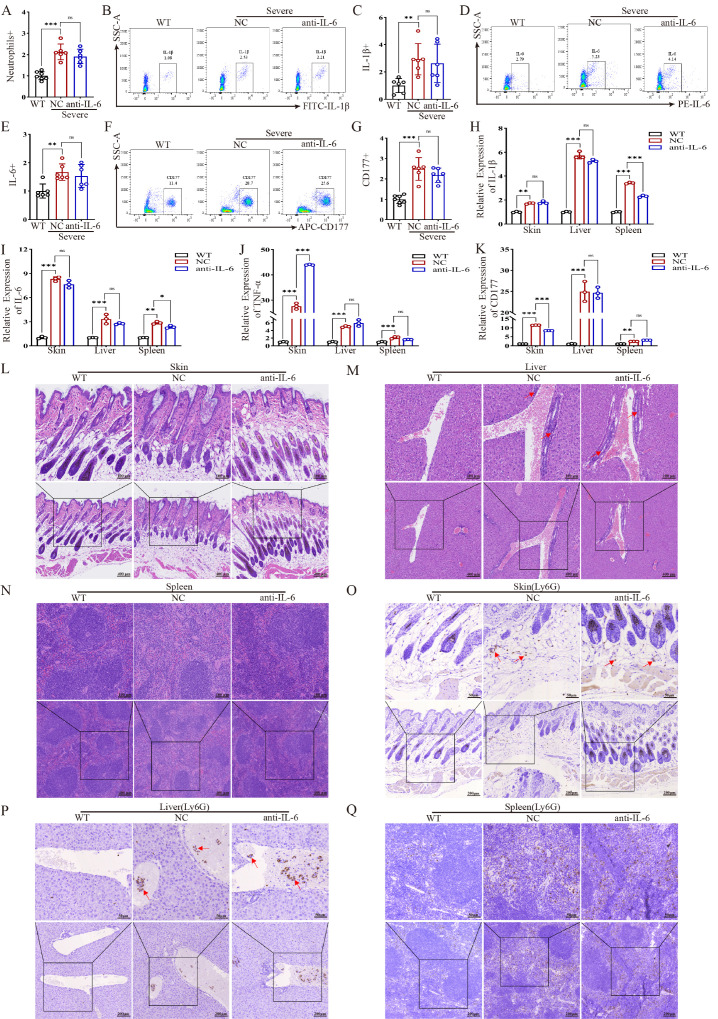
Targeted IL-6 therapy is ineffective for *NLRP3* mutation-induced AID. (**A**–**G**) Flow cytometry detection of neutrophil, IL-1β, IL-6 and CD177 protein expression in peripheral blood of NLRP3 mutant severe mice treated with or without IL-6 antibody (*n* = 7). (**H**–**K**) qPCR detection of IL-1β, IL-6, TNFα and CD177 mRNA expression levels in skin, liver, and spleen tissues of NLRP3 mutant severe mice treated with or without IL-6 antibody (*n* = 3). (**L**–**N**) Histopathological detection of mouse tissues (skin, liver, spleen), Scale bars: 400 μm, 100 μm. (**O**–**Q**) Immunohistochemical detection of Ly6G levels in mouse skin, liver, spleen tissues, Scale bars:200 μm, 50 μm. qPCR Data are shown as mean ± s.d. Student’s *t*-test was used. Statistical significance was determined as * *p* < 0.05; ** *p* < 0.01; *** *p* < 0.001; ns, not significant.

Given the pronounced elevation of IL-6, we evaluated the therapeutic potential of IL-6 blockade. Treatment with an IL-6 monoclonal antibody did not significantly reduce the elevated neutrophil count nor the aberrant expression of IL-1β, IL-6, or CD177 in the peripheral blood of severe mice (Figure 4A–C). qPCR analysis of tissues likewise showed no significant reduction in Il1b, Il6, Tnf, or Cd177 mRNA levels (Figure 4D–F).

Consistently, histopathological assessment (H&E staining) indicated only moderate improvement in skin inflammation, with persistent and marked inflammatory lesions in the liver and spleen of the treated group (Figure 4G–I). IHC analysis further confirmed that Ly6G+ neutrophil infiltration in these organs was not significantly reduced by IL-6 antibody treatment (Figure 4L–N). These results demonstrate the limited efficacy of IL-6 blockade in this model of severe NLRP3-AID.

### 2.4. Targeting CD177 Reverses the Inflammatory Phenotype and Shows Efficacy Comparable to IL-1β Blockade

To directly assess the therapeutic potential of targeting CD177, we treated severe mutant mice with CD177-specific siRNA, using an IL-1β antibody as a positive control. Prior to treatment, we confirmed no sex-based differences in the expression of key inflammatory markers (Supplementary Figure S1).

**Figure 5:**
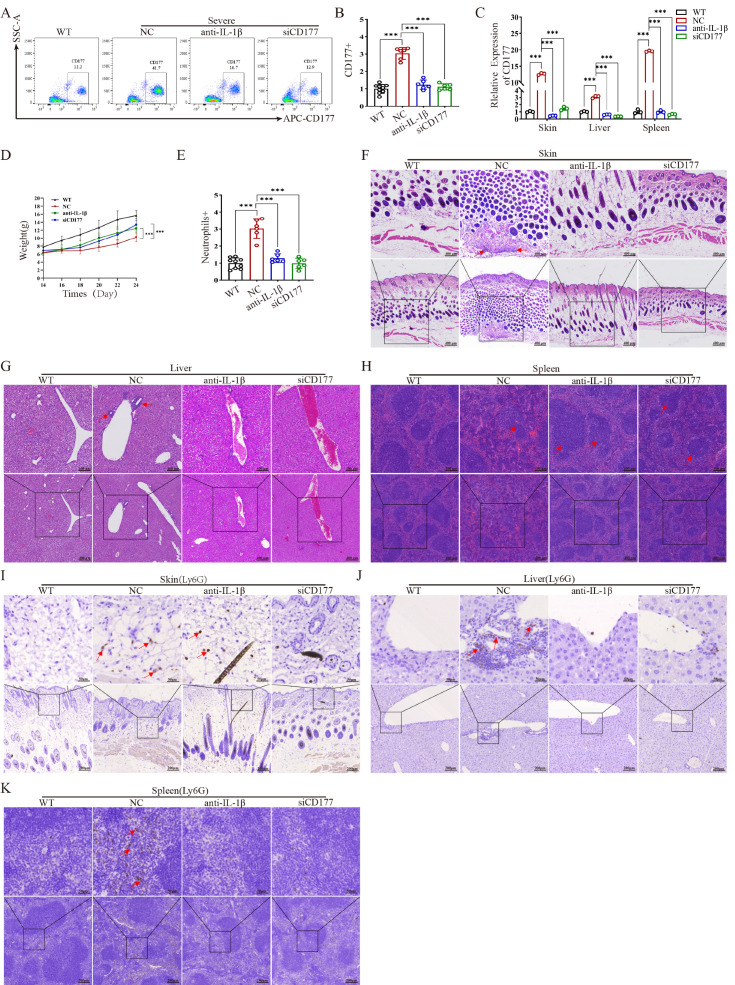
Targeting CD177 reverses the inflammatory phenotype of *NLRP3*-AID mice. (**A**,**B**) Flow cytometry detection of CD177 protein expression in peripheral blood of mice treated with either IL-1β antibody or CD177 siRNA (*n* = 6). (**C**) qPCR detection of CD177 mRNA expression levels in mouse skin, liver, and spleen tissues (*n* = 3). (**D**) Treatment was initiated on Day 14. Body weights were recorded every two days to evaluate systemic recovery. Data are presented as mean ± SEM (*n* = 6 per group). (**E**) Flow cytometry detection of neutrophils in mouse peripheral blood after treatment with either IL-1β antibody or CD177 siRNA (*n* = 6). (**F**–**H**) Histopathological detection of mouse tissues (skin, liver, spleen), Scale bars: 400 μm, 100 μm. (**I**–**K**) Immunohistochemical detection of Ly6G protein expression in mouse tissues (skin, liver, spleen), Scale bars: 200 μm, 50 μm. qPCR data are shown as mean ± s.d. Student’s *t*-test was used. Statistical significance was determined as *** *p* < 0.001.

CD177 siRNA treatment efficiently knocked down Cd177 expression in peripheral blood (Figure 5A,B) and in all examined tissues (Figure 5C). Both CD177 siRNA and IL-1β antibody treatment led to a significant rebound in body weight (Figure 5D) and a reduction in peripheral neutrophilia (Figure 5E).

Strikingly, histopathological evaluation (H&E staining) revealed that CD177 silencing significantly ameliorated inflammatory damage in the skin, liver, and spleen. The degree of recovery in the skin and spleen of the CD177 siRNA group was comparable to, and in some aspects potentially exceeding, that achieved with IL-1β blockade (Figure 5F–H). IHC analysis confirmed a significant reduction in Ly6G+ neutrophil infiltration in all target tissues following both treatments (Figure 5I–K).

### 2.5. CD177 Knockdown Suppresses the Expression of IL-1β and Key Inflammatory Cytokines

**Figure 6:**
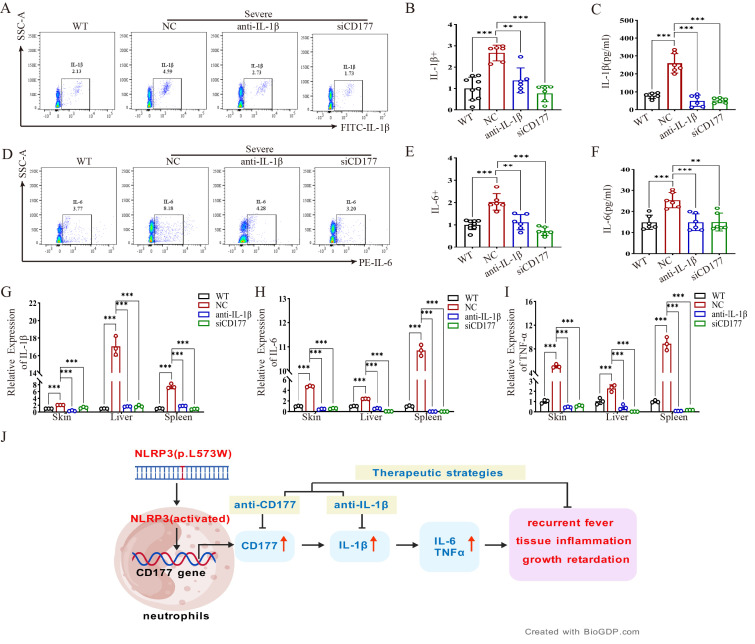
Targeting CD177 suppresses the expression of IL-1β. (**A**,**B**) Flow cytometry detection of IL-1β protein expression levels in peripheral blood of mice treated with either IL-1β antibody or CD177 siRNA (*n* = 6). (**C**) ELISA detection of serum IL-1β protein levels in mice treated with either IL-1β antibody or CD177 siRNA (*n* = 3). (**D**,**E**) Flow cytometry detection of IL-6 protein expression levels in peripheral blood of mice treated with either anti-IL-1β antibody or CD177 siRNA (*n* = 6). (**F**) ELISA detection of serum IL-6 protein levels in mice treated with either IL-1β antibody or CD177 siRNA (*n* = 3). (**G**–**I**) qPCR detection of IL-6, IL-1β, and TNFα mRNA expression levels in mouse skin, liver, and spleen tissues (*n* = 3). (**J**) Schematic diagram shows the role of CD177 in the development of NLRP3 mutation-related autoinflammation. qPCR Data are shown as mean ± s.d. Student’s *t*-test was used. Statistical significance was determined as ** *p* < 0.01; *** *p* < 0.001.

We further investigated the mechanism by which CD177 silencing ameliorates inflammation. Flow cytometry and ELISA analyses demonstrated that CD177 siRNA treatment significantly reduced the proportions of IL-1β^+^ and IL-6^+^ cells in peripheral blood and lowered serum levels of IL-1β and IL-6, with effects generally comparable to those of IL-1β antibody treatment (Figure 6A–F). Accordingly, qPCR confirmed that Il1b, Il6, and Tnf mRNA levels in the skin, liver, and spleen were markedly downregulated in the CD177 siRNA group (Figure 6G–I). A schematic summary illustrates the pivotal role of CD177 in amplifying NLRP3-driven inflammation (Figure 6J). Collectively, these results demonstrate that CD177, upregulated by hyperactive NLRP3 signaling, functions as a crucial driver of cytokine release and tissue inflammation. Targeting CD177 presents a novel and potent therapeutic strategy for *NLRP3*-AID.

## 3. Discussion

This study identifies *CD177* as a novel and critical downstream effector of NLRP3 inflammasome activation and a potent driver of pathological inflammation in NLRP3-AID. We demonstrate that gain-of-function mutations in *NLRP3* directly promote CD177 overexpression, which correlates with disease severity and amplifies a cascade of pro-inflammatory cytokines, including IL-1β, IL-6, and TNFα. Importantly, therapeutic targeting of CD177 via siRNA robustly ameliorated multi-organ inflammation and showed efficacy comparable to IL-1β blockade in our preclinical model, highlighting its potential as an alternative strategy for patients with suboptimal response to current biologics.

Our findings are grounded in clinical observations. Transcriptomic analysis of a patient carrying the *NLRP3* L573W mutation revealed CD177 as one of the most significantly upregulated genes, a finding validated at the protein level in peripheral blood. It should be noted that this initial discovery was based on a single patient, which is a limitation of our study. Future validation in larger cohorts is essential. This aligns with the established role of neutrophils as dominant effector cells in *NLRP3*-AID pathogenesis [23,24,25]. The specificity of this upregulation was confirmed through functional experiments in healthy human neutrophils, where NLRP3 agonism directly induced CD177 expression, an effect reversible by pathway inhibition. However, it is important to acknowledge that LPS, used for priming, activates multiple signaling pathways (e.g., NF-κB, MAPK). The use of the specific NLRP3 inhibitor CY-09, which reversed CD177 upregulation, provides strong evidence for the direct involvement of the NLRP3 inflammasome in this process. This positions CD177 not merely as a biomarker but as a specific downstream component of hyperactive NLRP3 signaling.

A significant challenge in studying NLRP3-AID has been modeling its clinical heterogeneity. The *NLRP3* L573W heterozygous mouse model we developed successfully recapitulates this spectrum, yielding both mild and severe inflammatory phenotypes. We observed that the absolute fold-change in inflammatory markers varied between independent experimental cohorts. Despite this numerical variability, which reflects the intrinsic biological heterogeneity and stochastic nature of autoinflammatory flares in the NLRP3 L573W model, the overall biological trends and statistical significance remained highly consistent across all experiments.

This heterogeneity allowed us to establish a strong correlation between CD177 expression levels and disease severity. Severe mice exhibited multi-organ pathology, profound neutrophilia, and elevated levels of IL-1β and IL-6, mirroring the human condition. This model thereby provides a robust platform for dissecting disease mechanisms and evaluating novel therapeutics.

A key finding of our study is the limited efficacy of IL-6 blockade despite its marked elevation in severe mice. Treatment with an IL-6 monoclonal antibody failed to significantly reduce neutrophilia, cytokine levels, or tissue pathology in key organs like the liver and spleen. This suggests that IL-6, while being a significant inflammatory marker, may function more as a downstream mediator rather than a primary driver in this specific NLRP3-mutant context. Its neutralization is insufficient to interrupt the core inflammatory cascade, underscoring the necessity of targeting upstream regulators.

The most compelling evidence for the therapeutic potential of CD177 comes from our intervention experiments. siRNA-mediated knockdown of CD177 significantly reversed the inflammatory phenotype, reducing neutrophil infiltration, suppressing IL-1β and IL-6 production, and facilitating structural recovery in the skin, liver, and spleen. When analyzed with appropriate multiple comparison tests, the therapeutic effects of CD177 silencing were found to be generally comparable in magnitude to those of IL-1β antibody treatment in our model. This aligns with and extends recent findings from a distinct inflammatory context, where CD177 was identified as a key mediator in acute respiratory distress syndrome (ARDS) by promoting neutrophil activation and NLRP3 inflammasome-driven inflammation [26]. The superiority of CD177 silencing over IL-1β antibody treatment suggests that its mechanism of action extends beyond simply inhibiting IL-1β. This efficacy suggests that CD177 and canonical cytokines form a self-amplifying positive feedback loop rather than a simple linear cascade. In this reciprocal network, CD177 drives cytokine production, while the resulting inflammatory milieu further augments CD177 expression, creating a ‘vicious cycle’ that sustains NLRP3-driven inflammation. By targeting CD177, we achieve a broader suppression of the inflammatory network than is possible with cytokine-specific blockade alone.

Our study has several limitations. The patient cohort is small, reflecting the rarity of NLRP3-AID. Future studies with larger, genetically diverse cohorts are needed to validate CD177 as a biomarker across different NLRP3 mutations. Furthermore, while we used an MCF-7 cell model to demonstrate the causal role of CD177 in cytokine regulation, future work should aim to elucidate the precise molecular mechanism by which CD177 modulates inflammation, potentially in more physiologically relevant cell systems. Notably, the basal expression of CD177 protein varies depending on the biological source, as seen in the inherent differences between human MCF-7 cells and healthy murine tissues. Despite these varying baselines, the consistent induction of CD177 upon NLRP3 activation across different models underscores its conserved role as a key inflammatory mediator. The exact mechanism by which CD177 regulates cytokine production remains unclear and is a subject for future investigation.

In conclusion, we have delineated a novel NLRP3-CD177-cytokine axis that drives pathology in NLRP3-AID. CD177 integrates upstream NLRP3 activation with downstream cytokine release, making it a superior therapeutic target compared to targeting individual cytokines. Our work provides a strong rationale for developing CD177-targeted therapies, which could offer new hope for patients refractory to existing treatments.

## 4. Materials and Methods

### 4.1. Ethics Statement

All animal experimental procedures in this study were approved by the Animal Experiment Ethics Committee of Guangdong Medical University (Approval No. GDY2502138, date: 6 January 2025) and conducted in accordance with the National Research Council’s Guide for the Care and Use of Laboratory Animals. Additionally, this study received ethical approval from the Ethics Committee of Dongguan Eighth People’s Hospital (Approval No. LL2023060903, date: 9 June 2023). Written informed consent was obtained from all participants prior to the commencement of the research.

### 4.2. RNA Sequencing

RNA sequencing was performed on peripheral blood samples collected from an NLRP3 p.Leu573Trp (L573W) patient and two age-matched healthy donors (designated as H1 and H2). Red blood cells were lysed using Erythrocyte Lysis Buffer (C3702, Beyotime, Shanghai, China), followed by TRIzol™ Reagent (15596018CN, Invitrogen, Carlsbad, CA, USA) treatment. All RNA samples were submitted to BGI-Shenzhen (Shenzhen, China) for high-throughput transcriptome sequencing. The raw sequencing data underwent quality control, alignment to the human reference genome (GRCh38), and quantification. Differential expression analysis was performed using DESeq2 to identify differentially expressed genes (DEGs) with a false discovery rate (FDR) < 0.05 and |log2(fold change)| > 1. Heatmap analysis was then conducted using GraphPad Prism 8.0 (GraphPad Software, San Diego, CA, USA).

### 4.3. Neutrophil Treatment Experiment

Neutrophils were isolated from the peripheral blood of healthy volunteers (designated as H3 and H4) using a commercial neutrophil isolation kit (TBD, LZS11131, Tianjin, China). To prime the NLRP3 inflammasome pathway, cells were first stimulated with 1 μg/mL lipopolysaccharide (LPS; Sigma-Aldrich, St. Louis, MO, L5293, USA) for 2 h. Following priming, neutrophils were treated for 1 h with either the NLRP3 activator nigericin (10 μM; Selleck, Houston, TX, E4788, USA) alone or co-treated with nigericin and the NLRP3 inhibitor CY-09 (10 μM; Selleck, Houston, TX, S5774, USA). Cells were then collected for downstream analysis.

LPS priming is a standard procedure to induce the transcriptional upregulation of NLRP3 and pro-IL-1β, thereby enabling subsequent inflammasome activation by a second signal. Thereafter, they were either treated with 10 μM Nigericin (E4788, Selleck, Houston, TX, USA) alone for 1 h or co-treated with 10 μM CY-09 (S5774, Selleck, Houston, TX, USA) for 1 h. Subsequently, the cells were harvested for subsequent experiments.

### 4.4. SiRNA Interference Assay

MCF-7 cells were seeded into 6-well plates at a density of 2 × 10^5^ cells per well. After 24 h, Lipofectamine 3000 transfection reagent (L3000015, Thermo Fisher, Waltham, MA, USA) was used to transfect negative control siRNA (NC) or CD177 siRNA (siCD177) into the cells according to the manufacturer’s instructions. The siRNA sequences are as follows: siCD177-1: sense 5′-GGACC ACCAUUAUGACACATT-3′, antisense 5′-UGUGUCAUAAUGGUGGUCCTT-3′; siCD177-2: sense 5′-CACCACGGCAAGUAUUUGUTT-3′, antisense 5′-ACAAAUACUUGCCGUGGUGTT-3′; NC: sense 5′-UUCUCCGAACGUGUCACGUTT-3′, antisense 5-ACGUGACACGUUCGG AGAATT-3′. Although MCF-7 cells are not of myeloid origin, they were chosen for initial in vitro functional validation due to their high transfection efficiency and low endogenous expression of innate immune sensors, which allows for a clearer assessment of the specific impact of CD177 modulation on cytokine expression without confounding signals from other pathways. For future studies, more physiologically relevant neutrophil-like cell lines (e.g., HL-60 or PLB985) will be employed.

### 4.5. Generation of the NLRP3 L573W Mutant Mouse Model

The *NLRP3* point mutation mouse model was engineered via CRISPR/Cas9 gene editing technology, with strain development conducted by GemPharmatech Co., Ltd. (Nanjing, Jiangsu, China). A sgRNA targeting exon 3 of the *NLRP3* gene (NCBI Gene ID: 216799; chromosomal location: Chr11: 59,345,672–59,370,981) was designed to introduce a c.1718T > G (p.Leu573Trp) missense mutation through homologous recombination, converting codon 573 from CTG (leucine) to TGG (tryptophan).

### 4.6. Enzyme-Linked Immunosorbent Assay (ELISA)

Mice were anesthetized by intraperitoneal injection of the anesthetic Avertin (JX750100, Genxion, Shanghai, China), and whole blood was collected by retro-orbital venous plexus puncture. After standing at room temperature for 2 h, the blood was centrifuged at 3000× *g* for 15 min at 4 °C to obtain serum. The levels of IL-1β and IL-6 in the serum were detected using mouse-specific IL-6 (CSB-E04639m, Cusabio, Wuhan, China) and IL-1β (CSB-E08054m, Cusabio, Wuhan, China) detection kits according to the manufacturer’s instructions.

### 4.7. qPCR Assay

RNA was extracted using an RNA extraction kit (EZB-RN4, EZB, Suzhou, China), followed by cDNA synthesis with a reverse transcription kit (A0010CGQ, EZB, Suzhou, China). Quantitative real-time PCR was performed using SYBR Green qPCR Master Mix (A0012, EZB, Suzhou, China) on a PCR instrument (Roche cobas Z 480, Rotkreuz, Switzerland). The relative mRNA expression levels were calculated using the *2*^−ΔΔCt^ method. For tissue-specific analysis, the expression levels in the experimental groups were normalized to the mean value of the control group (WT) for each respective tissue (skin, liver, or spleen) to determine the relative fold-change. Primer sequences were listed in Table 1.

### 4.8. Flow Cytometry Assay

Collect 100 μL of human or murine peripheral blood. Lyse red blood cells using a red blood cell lysis solution (349202, BD Biosciences, Franklin Lakes, NJ, USA). After incubating with primary antibodies for 1 h, wash the samples with PBS and then resuspend them. Subsequently, analyze the expression intensity of relevant proteins using a flow cytometer (BD FACSAria, BD Biosciences, San Jose, CA, USA). The details of the primary antibodies are as follows: murine CD177 antibody (566599, BD Biosciences, Franklin Lakes, NJ, USA), murine IL-6 antibody (504504, BioLegend, San Diego, CA, USA), murine IL-1β antibody (11-7114-82, Invitrogen, Carlsbad, CA, USA), and human CD177 antibody (315804, BioLegend, San Diego, CA, USA).

### 4.9. Western Blotting

Protein samples were isolated using RIPA lysis buffer supplemented with 1% protease inhibitor (A8260, Solarbio, Beijing, China). Following quantification by a BCA kit (P0010, Beyotime, Shanghai, China), the samples underwent 10% SDS–PAGE electrophoresis and were then transferred onto PVDF membranes (ISEQ00010, Millipore, Burlington, MA, USA). The membranes were blocked with 5% skim milk at room temperature for 1 h. Subsequently, they were incubated with primary antibodies at 4 °C overnight, followed by incubation with secondary HRP-conjugated antibodies at room temperature for 1 h. Finally, chemiluminescent signals (ECL) were captured using an imaging system (ChemiScope3600, Qinxiang, Shanghai, China). The details of the antibodies are as follows: CD177 (sc-376329, Santa Cruz Biotechnology, Santa Cruz, CA, USA), GAPDH (SC-137179, Santa Cruz Biotechnology, Santa Cruz, CA, USA), β-actin (sc-58673, Santa Cruz Biotechnology, Santa Cruz, CA, USA), HRP-conjugated Mouse antibody (K-20002M, Solarbio, Beijing, China), HRP-conjugated Rabbit antibody (SPA-238, Solarbio, Beijing, China).

### 4.10. Hematoxylin-Eosin Staining

After tissue collection from mice, the mouse specimens were immediately fixed in 4% paraformaldehyde (24197555, BioSharp, Hefei, China), followed by ethanol dehydration and paraffin embedding. Tissue blocks were sectioned into 4 μm slices using a microtome. After deparaffinization, hematoxylin and eosin (H&E) staining was performed using a commercial kit (C0105S, Beyotime, Shanghai, China). Stained sections were dehydrated and mounted with neutral resin for histopathological evaluation.

### 4.11. Immunohistochemistry Assay

For immunohistochemistry (IHC), the IHC Kit (SA1020, Boster Biological Technology, Wuhan, China) was utilized. Antigen retrieval was conducted using citrate buffer (pH 6.0), followed by quenching of endogenous peroxidase activity with 3% hydrogen peroxide. Non-specific binding was blocked with 10% goat serum. Sections were incubated with primary antibodies overnight at 4 °C, then probed with species-matched HRP-conjugated secondary antibodies. The sections were stained with the DAB kit (AR1027, Boster Biological Technology, Wuhan, China), dehydrated, and sealed with neutral resin for analysis.

### 4.12. Cytokines Microarray Assay

Following anesthesia, blood samples were collected from mice via retro-orbital plexus puncture and allowed to clot at room temperature for 2 h. Samples were centrifuged at 3000× *g* for 15 min at 4 °C. Serum specimens were transported on dry ice to Guangzhou Juyan Biotechnology Co., Ltd. (Guangzhou, China), for downstream analysis. Hierarchical clustering heatmap analysis was performed using GraphPad Prism 8.0 (GraphPad Software, San Diego, CA, USA).

### 4.13. Animal Treatment Experiment

Both male and female mice were used in this study. Preliminary assessments revealed no significant sex-specific differences in inflammatory markers (Supplementary Figure S1); therefore, data from both sexes were pooled for subsequent analyses, with *n* = 6 mice per group. Starting from Day 14, mice were subjected to in vivo therapeutic interventions using targeted antibodies and siRNA. To monitor the therapeutic response and systemic growth, body weights were recorded every two days. The administration protocols were as follows: Anti-IL-6 (A2118, Selleck, Houston, TX, USA) was administered at a dose of 10 mg/kg via intraperitoneal injection every three days for a total of four doses. Anti-IL-1β (BE0246, BioXcell, Lebanon, NH, USA) was administered at 10 mg/kg via intraperitoneal injection every three days, with four doses. siCD177 (GeneMedi, Shanghai, China) was administered at 5 mg/kg via intraperitoneal injection every three days, with four doses in total. The siRNA sequence for mouse CD177 was 5′-GGAUCAUCUCUGAUCUGAATT-3′ (sense) and 5′-UUCAGAUCA GAGAUGAUCCTT-3′ (antisense).

### 4.14. Statistical Analysis

Data are expressed as mean  ±  SEM. Statistical significance was determined by Student’s *t*-test (two groups) or one-way ANOVA followed by Tukey’s post hoc test for multiple comparisons (multiple groups). Graphs were generated with GraphPad Prism 8. Significance thresholds: * *p* < 0.05; ** *p* < 0.01; *** *p* < 0.001; ns (*p* > 0.05): not significant.

## Figures and Tables

**Table 1:** Primer sequences.

Gene	Forward Primer (5′→3′)	Reverse Primer (5′→3′)
CD177 (m)	GAGGGTTGCCAAGACTTGATAA	TGCTGTTCACATCATTGCAGAG
IL-6 (m)	CTGCAAGAGACTTCCATCCAG	AGTGGTATAGACAGGTCTGTTGG
IL-1β (m)	GAAATGCCACCTTTTGACAGTG	TGGATGCTCTCATCAGGACAG
TNFα (m)	CAGGCGGTGCCTATGTCTC	CGATCACCCCGAAGTTCAGTAG
β-actin (m)	GTGACGTTGACATCCGTAAAGA	GCCGGACTCATCGTACTCC
CD177 (h)	AAGAGATTACCAGCCACAGAC	GCTGAACTGTCCCAAACTG
IL-6 (h)	ACTCACCTCTTCAGAACGAATTG	CCATCTTTGGAAGGTTCAGGTTG
IL-1β (h)	ATGATGGCTTATTACAGTGGCAA	GTCGGAGATTCGTAGCTGGA
TNF-α (h)	AGCCCATGTTGTAGCAAACC	TGAGGTACAGGCCCTCTGAT
β-actin (h)	CATGTACGTTGCTATCCAGGC	CTCCTTAATGTCACGCACGAT
IL-18 (h)	TCTTCATTGACCAAGGAAATCGG	TCCGGGGTGCATTATCTCTAC

## Data Availability

The original contributions presented in this study are included in the article/Supplementary Material. Further inquiries can be directed to the corresponding author.

## Supplementary Materials

The following supporting information can be downloaded at: https://www.mdpi.com/article/10.3390/ijms27062841/s1.
