# Supplementary material for: Targeting CD177: A Novel Therapeutic Strategy for NLRP3-Associated Autoinflammatory Diseases
Source: Int J Mol Sci. 2026 Mar 20;27(6):2841. doi: 10.3390/ijms27062841 (PMC13026191; doi:10.3390/ijms27062841)
Supplement: Supplementary file 1 [file ijms-27-02841-s001.zip › ijms-4198618-supplementary.pdf]

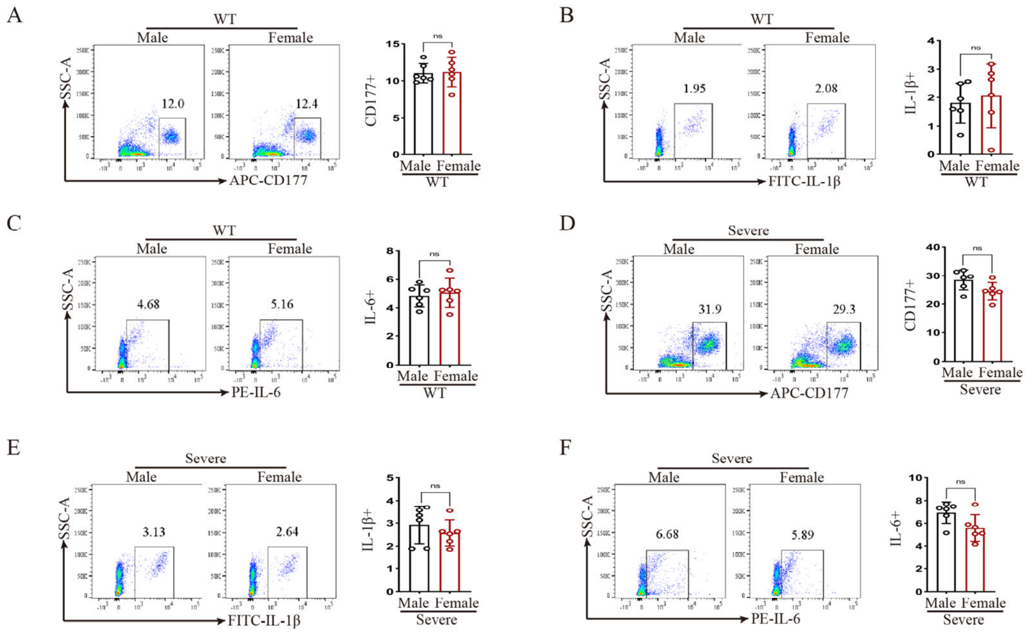

Supplementary Figure S1. Absence of Sex-Specific Differences in NLRP3-AID mice. (A-C) Flow cytometry detection of CD177, IL-1β and IL-6 expression in wild type male and female mice (n=6). (D-F) Flow cytometry detection of CD177, IL-1β and IL-6 expression in male and female mice with severe inflammation (n=6). qPCR Data are shown as mean±s.d. Student's t-test was used, Statistical significance was determined as *p < 0.05; **p < 0.01; ***p < 0.001.*p < 0.05; **p < 0.01; ***p < 0.001.
